# Evansville Medical School and Journal

**Published:** 1854-11

**Authors:** 


### EVANSVILLE MEDICAL SCHOOL AND JOURNAL.

A letter received from a professor in the Evansville Medical School and one of the editors of the *Indiana Journal of Medicine*, announces that the Faculty have "suspended the College operations and abandoned the Journal."

